# Cancer survivors’ experiences with mHealth interventions for PA: a meta-synthesis of qualitative studies

**DOI:** 10.3389/fpubh.2025.1715103

**Published:** 2025-12-03

**Authors:** Min Zhang, Jiashuai Chu, Wenzhe Sheng, Jing Bai, Yilin Song, Lei Fan

**Affiliations:** 1 Discipline Inspection Commission Office, The Third Affiliated Hospital of Heilongjiang University of Chinese Medicine, Harbin, China; 2 Graduate School of Heilongjiang University of Chinese Medicine, Harbin, China; 3 Xinglin College of Liaoning University of Traditional Chinese Medicine, Shenyang, Liaoning, China; 4 Department of Gastrointestinal Surgery, The Third Affiliated Hospital of Heilongjiang University of Chinese Medicine, Harbin, China

**Keywords:** cancer survivors, mHealth, PA, experience, qualitative meta-synthesis

## Abstract

**Background:**

Global cancer is rising, and many survivors struggle to stay active despite clear health benefits. Mobile health tools can help with tracking and guidance, but users’ real needs and barriers are unclear. This qualitative metasynthesis summarizes what survivors want, what helps, and what gets in the way.

**Methods:**

We systematically searched CNKI, Wanfang, VIP, CBM, PubMed, Cochrane Library, Embase, Web of Science, and CINAHL from inception to April 2025. Qualitative studies (or qualitative components of mixed-methods studies) exploring survivors’ experiences with mHealth-supported PA were eligible. Two reviewers independently screened studies, extracted data, and appraised methodological quality using the JBI qualitative checklist (2016). Findings were integrated via meta-synthesis, and confidence in synthesized findings was graded with the ConQual approach. Reporting followed ENTREQ.

**Results:**

Twelve qualitative studies (*n* = 243 participants; 8 countries) were included. Fifty findings were aggregated into 11 categories and synthesized into 3 higher-order findings: (1) survivors have multidimensional needs regarding content tailoring, professional input, social features, and usable design; (2) mHealth can strengthen motivation, self-efficacy, and convenience for engaging in PA; and (3) participation is shaped by patient-related, technology-related, and economic factors.

**Conclusion:**

Addressing survivors’ motivations, lived experiences, and practical barriers—through personalization, professional guidance, privacy-preserving social support, and robust technical design—may enhance adoption and sustained use of mHealth for PA in cancer survivorship.

**Systematic review registration:**

CRD420251140902.

## 1 Introduction

The International Agency for Research on Cancer (IARC) released global cancer statistics derived from the Global Cancer Observatory (GLOBOCAN), which compiles population-based data from 185 countries and territories. Drawing on temporal-trend projections, the global cancer burden in 2022 was estimated at ~20 million new cases and 9.7 million deaths. By 2050, incident cases are projected to reach 35.3 million—a 76.6% increase from 2022—while deaths are expected to rise to 18.5 million (an 89.7% increase). Together, these trends will nearly double the global cancer burden (1). In China, the National Cancer Center (NCC) estimated 4.82 million new cases and 2.57 million deaths in 2022, drawing on high-quality incidence and mortality data from 700 cancer registries nationwide (2).

Owing to advances in treatment and earlier detection, the five-year survival rate for patients with cancer has risen to ≈60% (3–5). Nonetheless, patients undergoing treatment face diverse challenges, including pain, fatigue, sensory disturbances, motor impairment, and weight gain (6–8). To improve quality of life, the American Cancer Society (ACS) Guideline on Nutrition and physical activity (PA) for Cancer Patients recommends 150–300 min of moderate-intensity or 75–150 min of vigorous-intensity PA per week (9). Moreover, evidence indicates that regular PA after a cancer diagnosis reduces all-cause and cancer-specific mortality and the risk of recurrence among survivors (10). Despite these benefits, a substantial proportion of cancer survivors remain inactive after treatment. Fewer than 10% maintain their activity levels during treatment, and only 20–30% remain active afterwardt (11).

The World Health Organization (WHO) defines mHealth as the use of portable wireless technologies—such as smartphones, wearables, and tablets—to expand access to health services (12). These platforms enable self-monitoring, activity tracking, and health education, supporting personalized physical-activity interventions that improve patient outcomes (13). Systematic reviews suggest that mHealth interventions can increase PA among cancer survivors (14). Although quantitative research demonstrates the effectiveness of mHealth in promoting PA among cancer survivors, qualitative studies are needed to elucidate survivors’ needs and the facilitators and barriers to mHealth use. Qualitative meta-synthesis aggregates findings across studies to evaluate survivors’ needs and experiences with mHealth-supported PA, providing a foundation for future research. Compared with prior work, our review updates the evidence base to April 2025, incorporates Chinese-language studies, and extends methodological coverage by offering actionable solutions to support implementation. Importantly, we move beyond description by proposing actionable strategies to support implementation. Compared with the 2022 quantitative systematic review, which focused on randomized controlled trials and effectiveness outcomes (14), this meta-synthesis centers on a qualitative perspective to explore in depth the unmet needs and contextual barriers of cancer survivors. It translates research findings into specific design recommendations and implementation pathways for mHealth tools, thereby bridging the evidence-to-practice gap.

## 2 Methods

### 2.1 Report guidelines

This systematic qualitative review used the Joanna Briggs Institute (JBI) metasynthesis methodology (15). Reporting followed the Enhancing Transparency in Reporting the Synthesis of Qualitative Research (ENTREQ) framework and addressed all 21 items (16) (see Supplementary material 1 for details). The protocol was prospectively registered with PROSPERO (CRD420251140902). We selected qualitative metasynthesis to derive interpretive insights beyond individual studies; searched Chinese- and English-language databases to mitigate language and publication bias; conducted dual independent screening with adjudication by a third reviewer to reduce selection bias; and used the JBI checklist and ConQual to appraise study quality and assess the confidence in synthesized findings.

### 2.2 Inclusion criteria

Inclusion criteria were specified according to the PICoS framework. (1) Participants (P): adults (≥18 years) who are cancer survivors. (2) Phenomenon of interest (I): perceptions and experiences of cancer survivors using moblie health (mHealth) (e.g., smartphones, tablets, wearable devices) to promote PA health at home or in healthcare settings. (3) Context (Co): use of mHealth at home or in healthcare settings. (4) Study design (S): qualitative research, including phenomenology, grounded theory, and ethnography. Mixed-methods studies were eligible if their qualitative components were reported separately.

### 2.3 Exclusion criteria

(1) Publications not in Chinese or English. (2) Publications without accessible full texts or with incomplete data. (3) Duplicate publications. (4) Reviews, case reports, and letters to the editor.

### 2.4 Search strategy

We searched PubMed, Cochrane Library, Embase, CINAHL, Web of Science, China National Knowledge Infrastructure (CNKI), VIP, Wanfang, and the China Biomedical Literature Database (CBM). Searches covered each database from inception to April 2025. Search terms included controlled vocabulary and keywords related to cancer (e.g., neoplasms, carcinoma, sarcoma, leukemia, lymphoma, cancer, tumor), mHealth (e.g., mHealth, app, mobile phone, activity monitor), PA (e.g., PA, exercise, physical exertion), and qualitative methods (e.g., qualitative research, ethnography, phenomenology, grounded theory, mixed methods, thematic analysis, descriptive research, interview). Full search strategies are provided in Supplementary material 2. PubMed, Embase, Cochrane Library, CINAHL, and Web of Science collectively cover biomedical literature, clinical trials and systematic reviews, nursing and allied health, and citation indexing, ensuring comprehensive retrieval on mHealth and PA. CNKI, Wanfang, VIP, and CBM were included to capture Chinese-language studies and grey literature underrepresented in international indexes, thereby reducing language and publication bias. Searching from inception facilitates identification of early and non-English studies and minimizes time-lag bias.

### 2.5 Literature screening

MZ and JC with formal training in evidence-based methods independently screened records and extracted data, then cross-checked the results. Records were imported into EndNote X9.1 for de-duplication, and duplicate publications were removed. Subsequently, titles and abstracts were screened to exclude studies that did not meet the inclusion criteria. Finally, full texts were assessed for eligibility to determine the final set of included studies. Discrepancies were resolved through discussion with a third reviewer. Data extraction captured author, year of publication, country, study design, participants, phenomenon of interest, and key findings. Study selection was documented using the PRISMA 2020 flow diagram, in accordance with PRISMA 2020.

### 2.6 Quality assessment

Two researchers independently appraised study quality using the 2016 JBI Critical Appraisal Checklist for Qualitative Research (15). Disagreements were resolved by discussion with a third reviewer. The checklist comprises 10 items, each rated “Yes,” “No,” “Unclear,” or “Not applicable.” Included studies were categorized into three grades: A (fully meeting criteria), B (partially meeting criteria), and C (not meeting criteria). Studies graded C were excluded. We used the JBI qualitative checklist because its domain-specific criteria are widely adopted for appraising qualitative evidence; excluding grade-C studies helps preserve the credibility of the synthesis.

### 2.7 Evaluation of meta-theme result quality

This study used a metasynthesis approach to integrate findings. Investigators, informed by the philosophical foundations and methods of major qualitative traditions, first extracted, translated, and interpreted the reported findings. They then analyzed themes and original statements, coded the data, synthesized similar findings into new categories, and examined relations among categories. Finally, these categories were synthesized into integrated findings. We used ConQual to rate the confidence in synthesized qualitative findings, providing a structured assessment of dependability and credibility for qualitative evidence. Each integrated finding was graded using ConQual (17). The ConQual system assesses five dependability domains and three credibility domains. For dependability, synthesized findings with 4–5 “Yes” responses were not downgraded; those with 2–3 were downgraded one level; those with 0–1 were downgraded two levels. For credibility, no downgrade occurred if all component findings were “Unequivocal”; a one-level downgrade applied if both “Unequivocal” and “Equivocal” were present; a two-level downgrade applied if all were “Equivocal”; a three-level downgrade applied if any were “Unsupported”; and a four-level downgrade applied if all were “Unsupported”.

## 3 Results

### 3.1 Search outcomes

**Figure 1:**
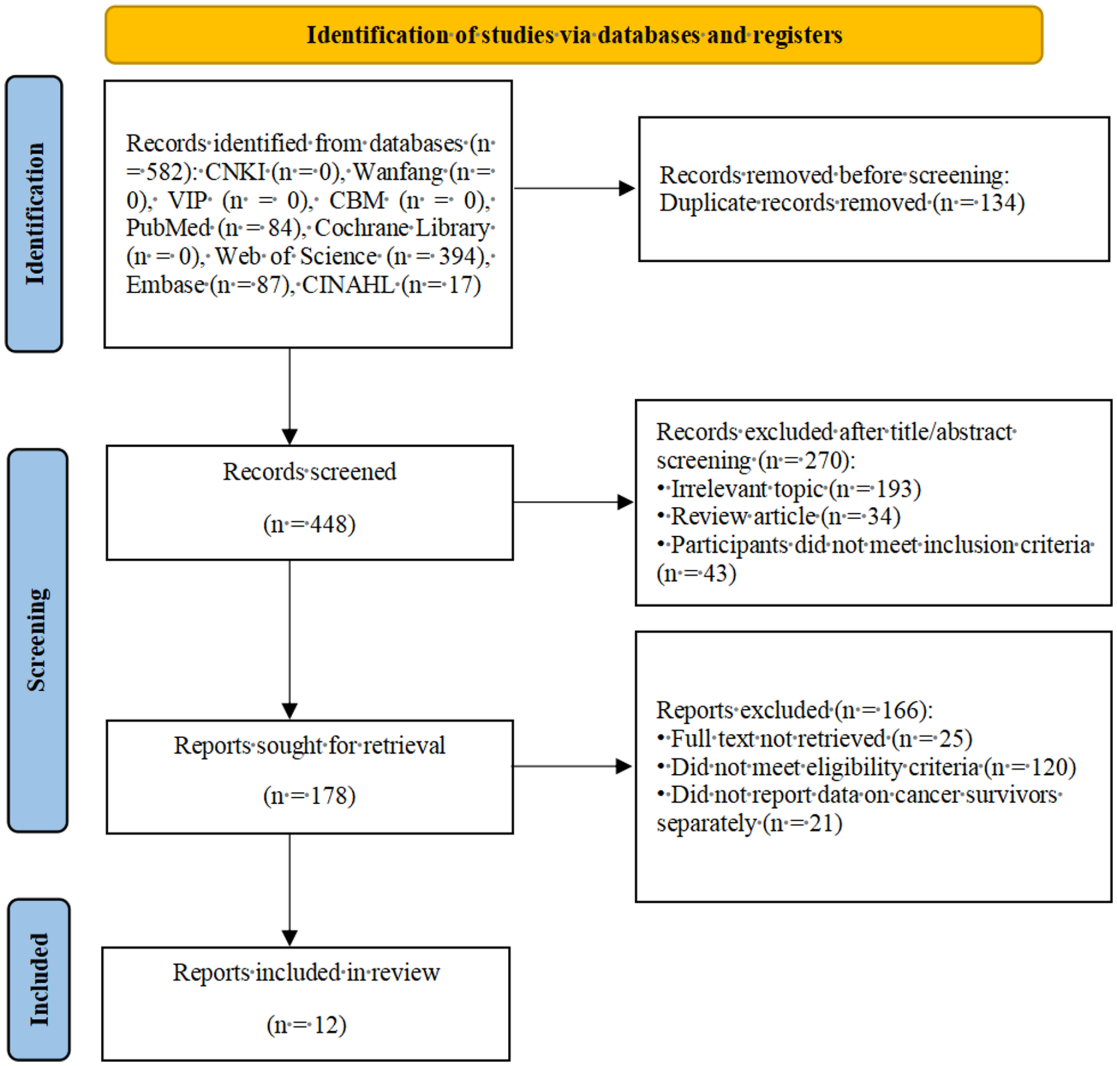
PRISMA 2020 flow diagram of study selection.

A total of 582 records were retrieved. After de-duplication in EndNote X9.1, 448 records remained. Screening of titles and abstracts excluded 270 records, leaving 178 for full-text review. Full-text assessment excluded 166 studies, yielding 12 included studies. The study-selection process is shown in Figure 1 (PRISMA 2020 flow diagram).

### 3.2 Characteristics of included studies

This review included 12 studies (18–29) comprising 243 cancer survivors across eight countries: United States (*n* = 4), United Kingdom (*n* = 2), China (*n* = 1), France (*n* = 1), Ireland (*n* = 1), Canada (*n* = 1), the Netherlands (*n* = 1), and Spain (*n* = 1). Baseline characteristics of the included studies are summarized in Table 1.

**Table 1:** Characteristics of included literatures (*n* = 12).

Author	Year	Country	Category	Participants	Phenomenon of interest	Themes extraction
Haberlin et al.	2020	Ireland	Thematic analysis	Cancer Survivors (*n* = 23)	Explore participants’ perspectives of eHealth enabled PA intervention	A1. Health Impact. A2. Education Needs. A3. Goal Setting. A4. Support Needs.
Porietis et al.	2019	Canada	Thematic analysis	Breast cancer survivors (*n* = 6)	To gain ‘perspectives on participation in a home-based PA intervention	B1. Study environment. B2. Influence of people. B3. Wearables
Lloyd et al.	2020	USA	Thematic analysis	Breast cancer survivors (*n* = 28)	Preferences for social support features in technology supported PA interventions	C1. Technology Increases Social Connectedness. C2. Professional Involvement and Support. C3. Connecting with Similar Survivors. C4. Apprehension Regarding Competitive Social Features.
Martin et al.	2021	France	Grounded theory	Breast cancer survivors (*n* = 9)	Perceptions of mHealth PA Interventions	D1. Levers to PA. D2. Barriers to PA. D3. Levers to mHealth Use. D4. Barriers to mHealth Use.
Guerra et al.	2020	Spain	Thematic analysis	Breast cancer survivors (*n* = 14)	Perspectives on PA Coaching apps	E1. Barriers to PA. E2. Psychological mediators of PA motivation. E3. Needs and suggestions for reinforcing motivation support. E4. Personalization aspects of the PA coaching experience. E5. Technology trustworthiness.
Nielsen et al.	2019	USA	Thematic analysis	Breast cancer survivors (*n* = 30)	Preferences for mHealth PA Interventions in	F1. Need for education about PA during chemotherapy. F2. Treatment side effects inhibit PA. F3. A structured, home-based, tech-supported program with in-person elements is most feasible. F4. Need for a personalized, highly tailored intervention. F5. Importance of social support from other breast cancer survivors, friends, and family.
Phillips et al.	2019	USA	Thematic analysis	Breast cancer survivors (*n* = 28)	Preferences for mHealth PA Interventions	G1. Importance of relevance to breast cancer survivors. G2. Easy to use. G3. Integration with wearables. G4. Encouraging and provide sense of accomplishment. G5. Level of structure and personalization.
Puszkiewicz et al.	2016	UK	Thematic analysis	cancer survivors (*n* = 11)	The Experiences of Using PA Mobile Applications	H1. Barriers to PA. H2. Receiving advice about PA from reliable sources. H3. Tailoring the application to one’s lifestyle. H4. Receiving social support from others.
Roberts et al.	2019	UK	Thematic analysis	Breast, Prostate, and Colorectal Cancer Survivors (*n* = 32)	The Experience of Using PA Mobile Applications	I1. The advantages and disadvantages of using apps to support PA. I2. The relevance of the app to the user. I3. The quality of the app. I4. The behavior change techniques used to promote PA.
Robertson et al.	2017	USA	Thematic analysis	cancer survivors (*n* = 35)	Mobile Health PA Intervention Preferences in	J1. A casual, concise, and positive tone. J2. Tools for personal goal attainment. J3. A prescription for PA. J4. An experience that is tailored to the user.
Wu et al.	2019	Netherlands	Thematic analysis	Breast Cancer Survivors (*n* = 10)	The experience of using activity trackers.	KI. The Activity Tracker and Accompanying app Raises Lifestyle Awareness. K2. Patients Need Personalized Advice. K3. Patients Need Personalized Advice. K4. Patients Need More Integration Between the Intervention Components of the Study
Yan et al.	2023	China	Constructivist qualitative study	Head and neck cancer (*n* = 17)	Exploring the Needs of Patients with HNC Regarding mHealth Based PA Interventions	L1. Functionality needs. L2. System usage requirements. L3. Social support. L4.perceived barriers and facilitators.

PA, physical activity; mHealth, mobile health; apps, mobile applications.

### 3.3 Quality appraisal

Using the JBI Qualitative Assessment and Review Instrument (JBI-QARI), we appraised the quality of the 12 included studies: 2 were graded A and 10 were graded B (Table 2).

**Table 2:** Quality assessment of included studies (*n* = 12).

Author	Q1	Q2	Q3	Q4	Q5	Q6	Q7	Q8	Q9	Q10	Quality
Haberlin et al	Yes	Yes	Yes	Yes	Yes	Yes	Yes	Yes	Yes	Yes	A
Porietis et al	Yes	Yes	Yes	Yes	Yes	No	No	Yes	Yes	Yes	B
Lloyd et al	Yes	Yes	Yes	Yes	Yes	No	No	Yes	Yes	Yes	B
Martin et al	Yes	Yes	Yes	Yes	Yes	No	No	unclear	Yes	Yes	B
Guerra et al	Yes	Yes	Yes	Yes	Yes	No	No	Yes	Yes	Yes	B
Nielsen et al	Yes	Yes	Yes	Yes	Yes	No	No	Yes	Yes	Yes	B
Phillips et al	Yes	Yes	Yes	Yes	Yes	No	No	Yes	Yes	Yes	B
Puszkiewicz et al	Yes	Yes	Yes	Yes	Yes	No	unclear	Yes	Yes	Yes	B
Roberts et al	Yes	Yes	Yes	Yes	Yes	Yes	No	Yes	Yes	Yes	B
Robertson et al	Yes	Yes	Yes	Yes	Yes	No	No	Yes	Yes	Yes	B
Wu et al	Yes	Yes	Yes	Yes	Yes	No	No	Yes	Yes	Yes	B
Yan et al	Yes	Yes	Yes	Yes	Yes	Yes	Yes	Yes	Yes	Yes	A

JBI QARI appraisal instruments: Q1. Is there congruity between the stated philosophical perspective and the research methodology? Q2. Is there congruity between the research methodology and the research question or objectives? Q3. Is there congruity between the research methodology and the methods used to collect data? Q4. Is there congruity between the research methodology and the representation and analysis of data? Q5. Is there congruity between the research methodology and the interpretation of results? Q6. Is there a statement locating the researcher culturally or theoretically? Q7. Is the influence of the researcher on the research, and vice-versa, addressed? Q8. Are participants, and their voices, adequately represented? Q9. Is the research ethical according to current criteria or, for recent studies, and is there evidence of ethical approval by an appropriate body? Q10. Do the conclusions draw in the research report flow from the analysis, or interpretation, of the data?

### 3.4 Findings of the review

Following iterative reading and comparative analysis, the findings were organized into 11 new categories and synthesized into three overarching integrated findings (Table 3).

**Table 3:** Findings extracted from the included studies, categories and synthesized findings.

Findings	Categories	Synthesized findings	Design implications to be detailed based on category specifics.	Reference
A4. Support Needs E4. Personalization aspects of the PA coaching experience. F4. Need for a personalized, highly tailored intervention. G5. Level of structure and personalization J1. A casual, concise, and positive tone. J4. An experience that is tailored to the user K2. Patients Need Personalized Advic L1. Functionality needs.	Cater to individual preferences	Cancer survivors have multidimensional needs for mobile health solutions to promote physical activity.	Adaptive plans by cancer type, stage, and treatment side-effects; adjustable goals; user-set notification windows; context-aware tips	Haberlin et al. (18), Monteiro-Guerra et al. (22). Phillips et al. (24). Puszkiewicz et al. (25)
A2. Education Needs. F1. Need for education about physical activity during chemotherapy. F2. Treatment side effects inhibit physical activity. G1. Importance of relevance to breast cancer survivors.	Health Education Needs		Evidence-based micro-lessons; stage-specific ‘do/avoid’ cards; clinician-approved resource hub; onboarding tutorials for low digital literacy. Evidence-based micro-lessons; stage-specific ‘do/avoid’ cards; clinician-approved resource hub; onboarding tutorials for low digital literacy.	Haberlin et al. (18), Nielsen et al. (23), Monteiro-Guerra et al. (22), Phillips et al. (24), Puszkiewicz et al. (25), Martin et al. (21); Ning et al. (29)
B1. Study environment. D1. Levers to Physical Activity. G2. Easy to use. I4. The behavior change techniques used to promote PA. KI. The Activity Tracker and Accompanying App K3. Patients Need Personalized Advice.	Function Design		Auto activity recognition (e.g., yoga/housework); real-time feedback (heart rate/steps); default privacy controls; offline mode and battery-saving; clear, minimal UI	Haberlin et al. (18), Kokts-Porietis et al. (19), Lloyd et al. (20), Monteiro-Guerra et al. (22), Phillips et al. (24), Puszkiewicz et al. (25); Robertson et al. (27), Wu et al. (28)
C1. Technology Increases Social Connectedness. C3. Connecting with Similar Survivors. D3. Levers to mHealth Use. F5. Importance of social support from other breast cancer survivors, friends, and family H4. Receiving social support from others L3.social support.	Social Needs		Opt-in peer groups of similar survivors; moderated communities; lightweight nudges from family/friends; weekly check-ins to sustain engagement.	Lloyd et al. (20), Nielsen et al. (23), Haberlin et al. (18), Kokts-Porietis et al. (19), Martin et al. (21), Ning et al. (29)
C2. Professional Involvement and Support. D2. Barriers to Physical Activity. H2. Receiving advice about PA from reliable sources. H3. Tailoring the application to one’s lifestyle. J3. A prescription for PA.	The Needs of Professionals		Clinician co-design; prescription templates for physical activity; automated referral prompts; in-app evidence citations for key recommendations.	Monteiro-Guerra et al. (22), Phillips et al. (24), Roberts et al. (26)
A1. Health Impact. A3. Goal Setting. B2. Influence of people. E3. Needs and suggestions for reinforcing motivation support.	Enhance patients’ motivation to engage in physical activity	Mobile Health Promotes the Benefits of Physical Activity for Cancer Survivors	SMART goals with streaks; progress badges; values-aligned goal framing; just-in-time motivational messages during low-mood periods.	Haberlin et al. (18), Kokts-Porietis et al. (19), Monteiro-Guerra et al. (22)
E2. Psychological mediators of PA motivation. G4. Encouraging and provide sense of accomplishment. J2. Tools for personal goal attainment. KI. The Activity Tracker and Accompanying App Raises Lifestyle Awareness.	Enhancing Self-Efficacy		Personal dashboards with symptom-titrated targets; trend views vs. baseline; success reflection prompts after each session.	Kokts-Porietis et al. (19), Martin et al. (21), Phillips et al. (24), Roberts et al. (26)
B3. Wearable technology F3. A structured, home-based, tech-supported program with in-person elements is most feasible. G3. Integration with wearables. I2. The relevance of the app to the user. I3. The quality of the app.	Convenience and efficiency		Home-based, asynchronous coaching; video + slow-motion demos; appointment-aware scheduling; forum alternatives to in-person groups.	Puszkiewicz et al. (25), Nielsen et al. (23), Ning et al. (29), Roberts et al. (26)
D4. Barriers to mHealth Use E1. Barriers to PA. H1. Barriers to PA. I1. The advantages and disadvantages of using apps to support PA.	Technology-related (barriers to mHealth use)	Factors Influencing Patients’ Participation in Mobile Health Based Physical Activities	Symptom-aware pacing (fatigue-based progression); selector for movement restrictions (e.g., lymphedema); low-burden modes for return-to-work.	Haberlin et al. (18), Martin et al. (21), Monteiro-Guerra et al. (22), Phillips et al. (24)
E5. Technology trustworthiness K4. Patients Need More Integration Between the Intervention Components of the Study L2. System usage requirements.	Equipment factors		Multi-sensor redundancy; error flags with manual edits; extended battery options; swim-proof wearables; offline caching for poor networks.	Kokts-Porietis et al. (19), Martin et al. (21), Roberts et al. (26), Wu et al. (28), Ning et al. (29)
L4. Perceived barriers and facilitators	Economic factors		Low-data mode; Wi-Fi-first sync; transparent data-use alerts; device-loan programs for low-income users.	Ning et al. (29)

#### 3.4.1 Synthesized finding 1: needs and user experience

##### 3.4.1.1 Individual tailoring and personalization

Content should be tailored according to the patient’s level (e.g. “Each person’s situation is different; interventions must be tailored to individual needs.” “The application must consider age, cancer type, treatment stage, and physical limitations.” “The rehabilitation needs for colorectal cancer and breast cancer differ; the application should distinguish between cancer types and treatment methods.”) (18, 22, 25). Physical limitations should be accommodated (e.g., “The application adjusts training recommendations based on lymphedema status.”) (22). It can recommend which type of exercise the patient should do based on their real-time status (“Indoor exercises are recommended on rainy days.”) (22). A specific function of the application allows for customized display based on the patient’s personal preferences (“It would be better if this function could be customized to show or hide.”) (24). Meanwhile, patients also hope to customize the application in their own way (“Hope to add a 30-min daily PA reminder.”) (25).

##### 3.4.1.2 Education and guidance needs

How patients should engage in proper PA and maintain a proper diet during treatment (“No one told me that exercise is important after treatment.” “During chemotherapy, I checked for side effects and dietary information every day, but the information available online varied greatly in quality.”) (18, 23). Timely update of information and professional advice (“Lack of guidance in the early stage.” “Many doctors still recommend plenty of rest, but patients may thus “rust” as a result.”) (22). Patients need exercise guidelines (“Doctors rarely discuss PA, and it is hoped that the application can fill this gap.”) (25). Provide usage guidance for patients during the initial use phase (“For people who are not familiar with technology, more detailed instructions are needed.” “We need someone specialized to teach us how to operate it.”) (21, 29).

##### 3.4.1.3 Functional design and usability

Patients need to receive feedback on their opinions in a timely manner (“It is hoped that feedback similar to that from researchers can be obtained every two weeks.”) (18). It can identify exercise modes and provide real-time feedback on exercise data (“view heart rate and step count data.” “Hope the device can recognize different types of activities, such as yoga and room cleaning, which consume different amounts of energy.”) (19, 24). A safe and trustworthy interactive online environment needs to be created (“On ordinary fitness apps, I do not know who the other users are, but here all users are patients with consistent goals”) (20). Meanwhile, patients hope to simplify the operation menu (“A simple and straightforward design is better,” “The interface should be clear at a glance,” “The app must be simple.”) (22, 24). Personal privacy is protected (“I do not want my exercise data to be accessed by other apps.” “The social module within the app should allow anonymous interaction to avoid privacy-related risks in interactions.”) (24, 25). The system’s voice reminders should be friendly, concise, humorous, and informal (“Avoid a clinical tone; it should be fun.” “If the message is too long, I might not want to read it.”) (27). Design interesting virtual characters (“Leveling up makes me feel amused.”) (22). Anthropomorphic interaction with virtual coaches (“A chat-style interface makes people feel like talking to a real coach.”) (22). Some patients believe that mHealth should expand its functions (“The app should integrate lifestyle recommendations, not just focus on PA.”) (25). Design a sedentary reminder function (“Vibration reminders make me aware of prolonged sitting.”) (28).

##### 3.4.1.4 Social support and community features

Patients hope that mHealth applications can have online social functions and allow them to share their own experiences (“When I finish exercising, I log into Facebook and post about it to share all my workouts together.” “If there is a community where fellow patients share exercise experiences, I will feel more motivated.”) (20, 23). Most patients hope to receive care (“I need to feel that someone cares about whether I am exercising” “The weekly email reminders make me feel noticed.”) (18, 19) and get support from family and friends (“Family and friends inviting me to go for a walk together makes me more motivated.” “The support from family members is crucial for overcoming barriers to exercise.”) (18, 29). Some other patients hope to interact with other patients (“I like group activities.”) (21).

##### 3.4.1.5 Professional involvement and evidence-based content

Regarding mHealth content sources, most patients prefer that healthcare professionals such as doctors and exercise specialists be involved in the design (“Seeing renowned hospitals participating in the development team makes me feel more confident using it” and “I would trust the app more if it had support from oncologists.”) (22). They also desire references to scientific evidence (“Providing support from medical literature makes me trust its training plans more”) (26). The specificity of intervention content (“Interventions must be tailored specifically to each patient… It is essential to know which exercises are suitable for postoperative recovery and which movements should be avoided.”) (24) enhances the professionalism and credibility of mHealth services, thereby safeguarding service quality.

#### 3.4.2 Synthesized finding 2: motivation and perceived benefits

##### 3.4.2.1 Motivation to engage in PA

Enhancing patient mobility (“Setting daily goals like walking distance motivates me to surpass previous records.” “I need to become healthier to face future challenges.”) (18, 19). Motivating myself to challenge preset goals (“The goals in the app make me want to push myself every day.” “This challenge is fantastic—it keeps driving me to surpass my limits.”) (18, 19). When cancer patients are in a negative state, building their confidence is crucial (“This requires a lot of effort. I feel deeply committed, and I believe I want to do this.”) (22). mHealth technology helps patients believe more in the benefits of PA, thereby motivating them (“It’s like having more strength and energy—that’s what drives me.”) (22).

##### 3.4.2.2 Self-efficacy and self-monitoring

Patients can objectively identify their own shortcomings, which helps them make changes (“Trackers make it easier for me to see my progress or shortcomings.”) (19), and enhances their self-monitoring ability (“Devices help me better understand my physical condition.”) (19). Patients develop good activity habits (“I maintain a daily goal of 6,000 steps.”) (21). Patients can intuitively see their own progress (“Seeing the animation of today’s goal being achieved makes me feel that my efforts have paid off.”) (24), which gives them a sense of satisfaction (“I feel a strong sense of accomplishment when I see the data meet or even exceed the target.”) (26).

##### 3.4.2.3 Convenience and access

mHealth demonstrates physical activities for patients to promote their progress (“The combination of video and voice guidance is very clear” “The slow-motion demonstration gives me time to adjust my posture.”) (25). The combination of mHealth technology and offline guidance brings a better experience to patients (“Having someone guide my exercise plan during IV infusion is not only convenient but also helps distract me from treatment anxiety.” “I barely go out in the first 10 days after chemotherapy, so home-based exercise is more suitable for me.” “Exercising at home avoids the embarrassment of being watched.”) (23, 29). Patients can participate even when they do not have time (“The built-in forum can replace offline support groups that are hard to attend.”) (25), which reduces time costs (“mHealth eliminates the trouble of traffic jams and parking.”) (29). The great convenience of mHealth technology brings a better experience to patients (“You can choose to exercise at any time—for example, if you want to move now, you can just open your phone and start.”) (26).

#### 3.4.3 Synthesized finding 3: determinants of participation

##### 3.4.3.1 Patient-related factors

Patients lose interest in physical activities after treatment (“After chemotherapy, I lost interest… and became extremely tired.”) (18) and have insufficient technical literacy (“It’s simply unrealistic to ask someone who has never used an app to use one directly.”) (18). Side effects caused after treatment (“After lymph node resection, the movement of my arm is limited.”) (21), lack of sufficient time (“I returned to full-time work immediately after treatment and really have no time to exercise.” “The pace of life is too fast, making time management difficult.”) (22), and advanced age (“I am 62 years old and find it very hard to learn new technologies.”) (24) are all factors that affect cancer patients’ participation in physical activities.

##### 3.4.3.2 Technology-related factors

Sometimes, device malfunctions prevent data recording or lead to inaccurate data logging (“I was clearly exhausted while rowing, but the device failed to record it.” “The device could not record data when I was cycling.”) (19, 21). Long-term use without updates makes patients feel bored (“I do not need the device to evaluate me anymore.”) (19). Poor internet access also causes issues (“If there’s no internet, my exercise records will be affected, and I will not know if I’ve met my PA goals.”) (29). Additionally, the battery life is too short (“I do not use it every day because it really drains your battery.”) (26), and the device cannot adapt to all types of patients’ exercises (“You cannot wear the tracker while swimming.”) (28).

##### 3.4.3.3 Economic factors

Patients worry about outdoor data charges (“My cellular data plan runs out easily, and exceeding it incurs extra fees.” and “Outdoor activities may cause data costs to exceed my budget.”) (29), which may lead some low-income patients to reduce their use of mHealth devices due to financial constraints.

### 3.5 Grading the certainty of evidence

We applied the ConQual approach to assess three synthesized findings; all were rated moderate (grade B), as shown in Table 4. For dependability, all synthesized findings met 4–5 “Yes” criteria and were not downgraded. For credibility, all synthesized findings were downgraded by one level, except “Factors Influencing Patients’ Participation in mHealth–Based Physical Activities,” which was unchanged. Overall, all three synthesized findings were rated moderate (grade B), indicating moderate credibility and dependability.

**Table 4:** ConQual summary of findings.

Synthesized finding	Type of research	Dependability	Credibility	ConQual score
Cancer survivors have multidimensional needs for mobile health solutions to promote physical activity.	Qualitative	No downgrade (2 studies scored 5/5 on all 5 criteria, 8 studies scored 4/5, and 1 studies scored 3/5)	Downgrade one level*	Moderate (B)
Mobile Health Promotes the Benefits of Physical Activity for Cancer Survivors	Qualitative	No downgrade (2 study scored 5/5 on all 7 criteria, 10 studies scored 4/5)	Downgrade one level *	Moderate (B)
Factors Influencing Patients’ Participation in Mobile Health Based Physical Activities	Qualitative	No downgrade (1 study scored 5/5 on all 5 criteria, 5 studies scored 4/5)	Remains unchanged	Moderate (B)

The symbol “*” indicates that the credibility of the corresponding synthesized finding was downgraded by one level during the ConQual evidence assessment. This downgrade is due to the presence of both “Unequivocal” and “Equivocal” component findings in the qualitative studies included in the synthesis, which aligns with the ConQual assessment criteria outlined in the manuscript.

## 4 Discussion

### 4.1 Prioritize patient needs and enhance user experience

Our findings show that cancer survivors’ needs for mHealth to support PA are heterogeneous, consistent with Vajravelu et al. (30). The likely explanations are as follows: (1) As mHealth technology evolves and functional capabilities expand, patients’ needs also shift. Therefore, a needs-driven approach should guide design, with continued expansion and refinement of mHealth functionality (31–34). (2) Emotional and tangible support from family, friends, and the wider community can reduce stress, sustain well-being, and improve quality of life (35–37). Accordingly, mHealth applications should incorporate forums, discussion groups, and related features to foster engagement, including participation by family members and peers. (3) As online content proliferates, patients can more readily access information—some accurate, some misleading. One survey reported that only 52% of clinicians were familiar with post-treatment PA guidelines, and many patients did not monitor PA in a timely manner (38). Consequently, many patients seek additional professional support to understand their condition and interpret guidance. This continued demand underscores the need for regularly updated mHealth platforms and content, making ongoing curation and evidence-based updates a priority for future development.

### 4.2 Strengthen patients’ motivation to exercise and enhance their perception of benefits

The findings of this study indicate that many patients derived positive experiences and regained a sense of self-worth when using mHealth to support physical activity (PA), consistent with Ghanbari et al. (39). Many cancer survivors report that mHealth tools enhance motivation for PA (40, 41), increase self-efficacy (42–44) and alleviate negative affect (45–47). These perceived physical and psychological benefits should be leveraged to motivate sustained PA, strengthen cognitive engagement with exercise, and foster habitual activity. Second, survivors may experience treatment-related changes (e.g., appearance, taste) that contribute to illness-related stigma (48, 49). mHealth provides a lower-burden, accessible complement to traditional interventions, reducing constraints related to transport, time, and location. Its efficiency and convenience can support survivors’ self-management (50, 51). mHealth technologies should be iteratively updated in response to patient feedback to better meet activity needs and enhance care.

### 4.3 Cancer patients also face multiple barriers

Our findings indicate that as mHealth technologies evolve and broaden their applications, user experience remains a primary determinant of adoption among cancer survivors, consistent with Park et al. (52). Patients may avoid or reduce mHealth use because of device–system incompatibilities and inaccurate activity recording (53, 54). In everyday life, treatment side effects, time constraints, and limited technical proficiency pose barriers during initial device use (55, 56). Accordingly, design should lower technical barriers through formative, user-centred research. Integrating AI-enabled voice interfaces can reduce operational burden and help patients use application features appropriately. Our synthesis suggests that core implementation principles—personalization, micro-education, social support, and clinician involvement—are highly transferable. Nevertheless, disease-specific nuances require attention. For example, post-surgical programs often require stricter safety rules and device workflows than routine oncology coaching; stroke and other neurologic conditions may necessitate accessibility-first UIs and caregiver-assisted onboarding to address digital-literacy and motor/cognitive limitations; and diabetes interventions frequently integrate dietary coaching and self-monitoring alongside activity goals.

### 4.4 Limitations

This metasynthesis of qualitative studies has limitations, and the findings should be interpreted with caution. First, of the 12 included studies, only two were graded A and the remaining ten were graded B. Moreover, most were conducted in cohorts of patients with breast cancer. This concentration may introduce bias and reduce the overall credibility of the synthesis. Second, all studies originated from eight countries that are predominantly high-income settings. As a result, generalizability to many countries is limited, which may constrain the applicability of the findings in other regions. Beyond these study-level limitations, mHealth approaches to promoting physical activity also face broader challenges. For example, accessibility barriers include limited clinical trial evidence and the absence of standardized user manuals for mHealth technologies. Technological literacy varies substantially across survivor populations. These factors may impede the widespread adoption of mHealth and prevent it from realizing its full potential.

## 5 Conclusion

This study uses a metasynthesis of qualitative research to examine cancer survivors’ experiences with mHealth-supported physical-activity interventions. It systematically identifies survivors’ needs and barriers in these interventions and delineates their benefits for survivors. The findings suggest that limited innovation and poor user adaptiveness in mHealth systems are associated with low motivation for physical activity, manifesting as sedentary behavior and reduced quality of life. Consistent with Maslow’s hierarchy of needs, higher-order motives become primary only after lower-order needs are met. Accordingly, safeguarding survivors’ physical and psychological needs is paramount. Family members also play a vital role: they can use mHealth to foster positive affect and support survivors’ reintegration into daily life.

## Data Availability

The original contributions presented in the study are included in the article/Supplementary material, further inquiries can be directed to the corresponding author.
