# Supplementary material for: Cancer survivors’ experiences with mHealth interventions for PA: a meta-synthesis of qualitative studies
Source: Front Public Health. 2025 Dec 3;13:1715103. doi: 10.3389/fpubh.2025.1715103 (PMC12708573; doi:10.3389/fpubh.2025.1715103)
Supplement: Supplementary file 2 [file Table_2.DOCX]

**Database Search Strategy**

**Take PubMed as an example:**

#1 ((((((Neoplasms[Title/Abstract]) OR (Carcinoma[Title/Abstract])) OR (Sarcoma[Title/Abstract])) OR (Leukemia[Title/Abstract])) OR (Lymphoma[Title/Abstract])) OR (Cancer[Title/Abstract])) OR (Tumor[Title/Abstract])

#2 ((((Mobile health[Title/Abstract]) OR (mHealth[Title/Abstract])) OR (APP[Title/Abstract])) OR (mobile phone[Title/Abstract])) OR (activity monitor[Title/Abstract])

#3 ((physical activity[Title/Abstract]) OR (Physical exertion[Title/Abstract])) OR (Exercise[Title/Abstract])

#4 ((((((((Qualitative research[Title/Abstract]) OR (qualitative study[Title/Abstract])) OR (ethnography[Title/Abstract])) OR (phenomenology[Title/Abstract])) OR (grounded theory[Title/Abstract])) OR (mixed methods[Title/Abstract])) OR (thematic analysis[Title/Abstract])) OR (descriptive research[Title/Abstract])) OR (Interview[Title/Abstract])

#5 #1 AND #2 AND #3 AND #4

**Take Web of Science as an example:**

#1Neoplasms (Topic) or Carcinoma (Topic) or Sarcoma (Topic) or Leukemia (Topic) or Lymphoma (Topic) or Cancer (Topic) or Tumor (Topic)

#2 Mobile health (Topic) or mHealth (Topic) or APP (Topic) or mobile phone (Topic) or activity monitor (Topic)

#3 physical activity (Topic) or exercise (Topic) or Physical exertion (Topic)

#4 Qualitative research (Topic) or qualitative study (Topic) or ethnography (Topic) or phenomenology (Topic) or grounded theory (Topic) or mixed methods (Topic) or thematic analysis (Topic) or descriptive research (Topic) or Interview (Topic)

#5 #1 AND #2 AND #3 AND #4

**Take CINAHL as an example:**

S1 [XB (Neoplasms) OR XB (Carcinoma) OR XB (Sarcoma) OR XB (Leukemia) OR XB (Lymphoma) OR XB (Cancer) OR XB (Tumor)](https://research.ebsco.com/search/results?db=ccm&expanders=concept&limiters=None&q=XB (Neoplasms) OR XB (Carcinoma) OR XB (Sarcoma) OR XB (Leukemia) OR XB (Lymphoma) OR XB (Cancer) OR XB (Tumor)&qm=W3sidmFsdWUiOiJOZW9wbGFzbXMiLCJ0eXBlIjoiZmllbGQiLCJjb2RlIjoiWEIifSx7InZhbHVlIjoiT1IiLCJ0eXBlIjoibG9naWMifSx7InZhbHVlIjoiQ2FyY2lub21hIiwidHlwZSI6ImZpZWxkIiwiY29kZSI6IlhCIn0seyJ2YWx1ZSI6Ik9SIiwidHlwZSI6ImxvZ2ljIn0seyJ2YWx1ZSI6IlNhcmNvbWEiLCJ0eXBlIjoiZmllbGQiLCJjb2RlIjoiWEIifSx7InZhbHVlIjoiT1IiLCJ0eXBlIjoibG9naWMifSx7InZhbHVlIjoiTGV1a2VtaWEiLCJ0eXBlIjoiZmllbGQiLCJjb2RlIjoiWEIifSx7InZhbHVlIjoiT1IiLCJ0eXBlIjoibG9naWMifSx7InZhbHVlIjoiTHltcGhvbWEiLCJ0eXBlIjoiZmllbGQiLCJjb2RlIjoiWEIifSx7InZhbHVlIjoiT1IiLCJ0eXBlIjoibG9naWMifSx7InZhbHVlIjoiQ2FuY2VyIiwidHlwZSI6ImZpZWxkIiwiY29kZSI6IlhCIn0seyJ2YWx1ZSI6Ik9SIiwidHlwZSI6ImxvZ2ljIn0seyJ2YWx1ZSI6IlR1bW9yIiwidHlwZSI6ImZpZWxkIiwiY29kZSI6IlhCIn1d&searchMode=boolean&sort=relevance&userDirectAction=true&isDashboardExpanded=true)

S2 [XB (Mobile health) OR XB (mHealth) OR XB (APP) OR XB (mobile phone) OR XB (activity monitor)](https://research.ebsco.com/search/results?db=ccm&expanders=concept&limiters=None&q=XB (Mobile health) OR XB (mHealth) OR XB (APP) OR XB (mobile phone) OR XB (activity monitor)&qm=W3sidmFsdWUiOiJNb2JpbGUgaGVhbHRoIiwidHlwZSI6ImZpZWxkIiwiY29kZSI6IlhCIn0seyJ2YWx1ZSI6Ik9SIiwidHlwZSI6ImxvZ2ljIn0seyJ2YWx1ZSI6Im1IZWFsdGgiLCJ0eXBlIjoiZmllbGQiLCJjb2RlIjoiWEIifSx7InZhbHVlIjoiT1IiLCJ0eXBlIjoibG9naWMifSx7InZhbHVlIjoiQVBQIiwidHlwZSI6ImZpZWxkIiwiY29kZSI6IlhCIn0seyJ2YWx1ZSI6Ik9SIiwidHlwZSI6ImxvZ2ljIn0seyJ2YWx1ZSI6Im1vYmlsZSBwaG9uZSIsInR5cGUiOiJmaWVsZCIsImNvZGUiOiJYQiJ9LHsidmFsdWUiOiJPUiIsInR5cGUiOiJsb2dpYyJ9LHsidmFsdWUiOiJhY3Rpdml0eSBtb25pdG9yIiwidHlwZSI6ImZpZWxkIiwiY29kZSI6IlhCIn1d&searchMode=boolean&sort=relevance&userDirectAction=true&isDashboardExpanded=true)

S3 [XB (physical activity) OR XB (Physical exertion) OR XB (Exercise)](https://research.ebsco.com/search/results?db=ccm&expanders=concept&limiters=None&q=XB (physical activity) OR XB (Physical exertion) OR XB (Exercise)&qm=W3sidmFsdWUiOiJwaHlzaWNhbCBhY3Rpdml0eSIsInR5cGUiOiJmaWVsZCIsImNvZGUiOiJYQiJ9LHsidmFsdWUiOiJPUiIsInR5cGUiOiJsb2dpYyJ9LHsidmFsdWUiOiJQaHlzaWNhbCBleGVydGlvbiIsInR5cGUiOiJmaWVsZCIsImNvZGUiOiJYQiJ9LHsidmFsdWUiOiJPUiIsInR5cGUiOiJsb2dpYyJ9LHsidmFsdWUiOiJFeGVyY2lzZSIsInR5cGUiOiJmaWVsZCIsImNvZGUiOiJYQiJ9XQ==&searchMode=boolean&sort=relevance&userDirectAction=true&isDashboardExpanded=true)

S4 XB (Qualitative research) OR XB (qualitative study) OR XB (ethnography) OR XB (phenomenology) OR XB (grounded theory) OR XB (mixed methods) OR XB (thematic analysis) OR XB (descriptive research) AND XB (Interview)

S5 [S1 AND S2 AND S3 AND S4](https://research.ebsco.com/search/results?combinedSearchQueryId=sq:cd7f98de-afdc-4d68-b6d5-6e4bd9cb42b5&db=ccm&expanders=concept&limiters=None&q=S1 AND S2 AND S3 AND S4&qm=W3sidmFsdWUiOiJzcTpkZmIwNjExMi1iYWVkLTQwY2ItYTNhNi1mMmI4OTNkODRmNWMiLCJ0eXBlIjoidGV4dCJ9LHsidmFsdWUiOiJBTkQiLCJ0eXBlIjoibG9naWMifSx7InZhbHVlIjoic3E6ZDA0NTI4YWUtMmVhOS00YzEzLThhNWYtMGU0OGVkNzQwYzQzIiwidHlwZSI6InRleHQifSx7InZhbHVlIjoiQU5EIiwidHlwZSI6ImxvZ2ljIn0seyJ2YWx1ZSI6InNxOmJhYTNlNGUxLTYxMzYtNDhmMS04NjQ2LTBhMjdiMjY4ZjY4YyIsInR5cGUiOiJ0ZXh0In0seyJ2YWx1ZSI6IkFORCIsInR5cGUiOiJsb2dpYyJ9LHsidmFsdWUiOiJzcTpiMDk3YzVkNi03OGVkLTQwZjktOTdlMC02OWJjNWE2MDU3ZDMiLCJ0eXBlIjoidGV4dCJ9XQ==&searchMode=boolean&sort=relevance&userDirectAction=true&isDashboardExpanded=true)

**Take Embase as an example:**

#1 'neoplasms':ab,ti OR 'carcinoma':ab,ti OR 'sarcoma':ab,ti OR 'leukemia':ab,ti OR 'lymphoma':ab,ti OR 'cancer':ab,ti OR 'tumor':ab,ti

#2 'mobile health':ab,ti OR 'mhealth':ab,ti OR ‘app’:ab,ti OR 'mobile phone':ab,ti OR 'activity monitor':ab,ti

#3 'physical activity':ab,ti OR 'exercise':ab,ti OR 'physical exertion':ab,ti

#4 'qualitative research':ab,ti OR 'qualitative study':ab,ti OR 'physical exertion':ab,ti OR ethnography:ab,ti OR phenomenology:ab,ti OR 'grounded theory':ab,ti OR 'mixed methods':ab,ti OR 'thematic analysis':ab,ti OR 'descriptive research':ab,ti OR 'interview':ab,ti

#5 #1 AND #2 AND #3 AND #4
